# Supplementary material for: Puerarin Induces Molecular Details of Ferroptosis-Associated Anti-Inflammatory on RAW264.7 Macrophages
Source: Metabolites. 2022 Jul 15;12(7):653. doi: 10.3390/metabo12070653 (PMC9317776; doi:10.3390/metabo12070653)
Supplement: Supplementary file 1 [file metabolites-12-00653-s001.zip › metabolites-1789307-supplementary.pdf]

Supplement file

2.2. Result of Network pharmacology

Supplement Table S1: The 51 potential anti-inflammatory targets of puerarin

Gene symbol	Uniprot ID	Protein name	Degree
Akt1	P31750	RAC-alpha serine/threonine-protein kinase	30
Jun	P05627	Transcription factor Jun	26
Stat3	P42227	Signal transducer and activator of transcription 3	24
Esr1	P19785	Estrogen receptor	20
Hsp90aa1	P07901	Heat shock protein HSP 90-alpha	20
Tnf	P06804	Tumor necrosis factor	18
Rela	Q04207	Transcription factor p65	16
Casp3	P70677	Caspase-3	12
Ar	P19091	Androgen receptor	10
Mapk9	Q9WTU6	Mitogen-activated protein kinase 9	10
Fos	P01101	Protein c-Fos	10
Esr2	O08537	Estrogen receptor beta	10
Ahr	**P30561**	**Aryl hydrocarbon receptor**	**8**
Bcl2	P10417	Apoptosis regulator Bcl-2	8
Hif1a	Q61221	Hypoxia-inducible factor 1-alpha	8
Bad	Q61337	Bcl2-associated agonist of cell death	8
Nos3	P70313	Nitric oxide synthase, endothelial	8
Prkaca	P05132	cAMP-dependent protein kinase catalytic subunit alpha	8
Cyp1a2	P00186	Cytochrome P450 1A2	6
Ptpn1	P35821	Tyrosine-protein phosphatase non-receptor type 1	6
Nos2	P29477	Nitric oxide synthase, inducible	6
Casp9	Q8C3Q9	Caspase-9	6
Gsk3b	Q9WV60	Glycogen synthase kinase-3 beta	6
Alox15	**P39654**	**Polyunsaturated fatty acid lipoxygenase ALOX15**	**6**
Casp8	O89110	Caspase-8	6
Pparg	P37238	Peroxisome proliferator-activated receptor gamma	6
Nfkbia	Q9Z1E3	NF-kappa-B inhibitor alpha	6
Tert	O70372	Telomerase reverse transcriptase	4
Birc5	O70201	Baculoviral IAP repeat-containing protein 5	4
Fas	P25446	Tumor necrosis factor receptor superfamily member 6	4
Lepr	P48356	Leptin receptor	4
Prkce	P16054	Protein kinase C epsilon type	4
Timp2	P25785	Metalloproteinase inhibitor 2	4
Adora2a	Q60613	Adenosine receptor A2a	2
Adora3	Q61618	Adenosine receptor A3	2
Cdkn1b	P46414	Cyclin-dependent kinase inhibitor 1B	2
Aldh2	P47738	Aldehyde dehydrogenase, mitochondrial	2
Maoa	Q64133	Amine oxidase [flavin-containing] A	2
Ptgs1	P22437	Prostaglandin G/H synthase 1	2

Gene symbol	Uniprot ID	Protein name	Degree
Ptgs2	**Q05769**	**Prostaglandin G/H synthase 2**	**2**
Bax	Q07813	Apoptosis regulator BAX	2
Cyp19a1	P28649	Aromatase	2
Xdh	Q00519	Xanthine dehydrogenase/oxidase	2
Dnmt1	P13864	DNA (cytosine-5)-methyltransferase 1	2
Nr2f2	P43135	COUP transcription factor 2	2
Ripk2	P58801	Receptor-interacting serine/threonine-protein kinase 2	2
Ifna1	P01572	Interferon alpha-1	2
Jak3	Q62137	Tyrosine-protein kinase JAK3	2
Mmp2	P33434	72 kDa type IV collagenase	2
Mmp9	P41245	Matrix metalloproteinase-9	2
Vcam1	P29533	Vascular cell adhesion protein 1	2

Supplement Table S2: A total of 136 KEGG pathways enriched by network pharmacology analysis

Term	pathway	PValue	Fold Enrichment	FDR
mmu05200	Pathways in cancer	9.73089E-34	20.48099675	8.85511E-32
mmu05417	Lipid and atherosclerosis	4.02068E-28	36.80396929	1.82941E-26
mmu05161	Hepatitis B	6.55286E-25	41.49211781	1.9877E-23
mmu05162	Measles	4.71186E-24	44.65639317	1.07195E-22
mmu04210	Apoptosis	4.66967E-19	39.92998659	8.49881E-18
mmu04933	AGE-RAGE signaling pathway in diabetic complications	5.70982E-19	49.57004161	8.65989E-18
mmu01522	Endocrine resistance	1.4596E-17	49.90547798	1.89748E-16
mmu05167	Kaposi sarcoma-associated herpesvirus infection	2.03694E-17	25.37000887	2.31702E-16
mmu05169	Epstein-Barr virus infection	2.47528E-17	25.0433564	2.50279E-16
mmu04668	TNF signaling pathway	1.41912E-16	41.58789832	1.2914E-15
mmu05207	Chemical carcinogenesis - receptor activation	6.89266E-16	23.88824525	5.70211E-15
mmu04657	IL-17 signaling pathway	8.85776E-16	46.55666522	6.71713E-15
mmu05170	Human immunodeficiency virus 1 infection	1.76735E-15	22.32819658	1.23714E-14
mmu05145	Toxoplasmosis	5.40134E-15	39.78478664	3.51087E-14
mmu04932	Non-alcoholic fatty liver disease	7.95908E-15	30.00645828	4.82851E-14
mmu05160	Hepatitis C	1.66958E-14	28.22035957	9.49571E-14
mmu05210	Colorectal cancer	3.12258E-14	45.07452419	1.6715E-13
mmu05152	Tuberculosis	4.9902E-14	25.76641526	2.52282E-13
mmu05215	Prostate cancer	1.27736E-13	39.32973189	5.97384E-13
mmu05163	Human cytomegalovirus infection	1.31293E-13	19.48745911	5.97384E-13
mmu05418	Fluid shear stress and	2.01725E-13	28.79162191	8.74142E-13

Term	pathway	PValue	Fold Enrichment	FDR
	atherosclerosis			
mmu04926	Relaxin signaling pathway	1.63423E-12	30.62315002	6.75979E-12
mmu04915	Estrogen signaling pathway	2.76319E-12	29.06980183	1.05839E-11
mmu05132	Salmonella infection	2.79137E-12	18.37604809	1.05839E-11
mmu05222	Small cell lung cancer	3.94347E-12	37.59730696	1.43542E-11
mmu04936	Alcoholic liver disease	4.2399E-12	27.85856009	1.48396E-11
mmu05142	Chagas disease	9.70006E-12	34.08353996	3.26928E-11
mmu05165	Human papillomavirus infection	1.04494E-11	13.7992278	3.39606E-11
mmu04659	Th17 cell differentiation	1.3563E-11	32.85530428	4.25598E-11
mmu04931	Insulin resistance	1.4719E-11	32.56195335	4.46478E-11
mmu04722	Neurotrophin signaling pathway	3.20342E-11	29.89294078	9.40359E-11
mmu04217	Necroptosis	3.71809E-11	22.4113556	1.05733E-10
mmu01524	Platinum drug resistance	5.4898E-11	39.07434402	1.51385E-10
mmu04215	Apoptosis - multiple species	2.84814E-10	77.35930736	7.49476E-10
mmu04620	Toll-like receptor signaling pathway	2.8826E-10	31.86645532	7.49476E-10
mmu05208	Chemical carcinogenesis - reactive oxygen species	3.68589E-10	17.75058696	9.3171E-10
mmu05203	Viral carcinogenesis	4.75942E-10	17.29152006	1.17056E-09
mmu05164	Influenza A	8.56245E-10	20.72124304	2.05048E-09
mmu05166	Human T-cell leukemia virus 1 infection	1.06315E-09	15.91917719	2.48068E-09
mmu04917	Prolactin signaling pathway	1.21486E-09	38.38882922	2.76381E-09
mmu04071	Sphingolipid signaling pathway	1.45427E-09	26.04956268	3.22778E-09
mmu05022	Pathways of neurodegeneration - multiple diseases	3.64598E-09	9.856591285	7.89963E-09
mmu05205	Proteoglycans in cancer	4.06043E-09	17.36637512	8.593E-09
mmu04621	NOD-like receptor signaling pathway	4.78976E-09	17.04176998	9.9061E-09
mmu05010	Alzheimer disease	5.22756E-09	11.19265097	1.05713E-08
mmu04660	T cell receptor signaling pathway	1.11579E-08	28.0533752	2.20732E-08
mmu04625	C-type lectin receptor signaling pathway	1.99206E-08	25.81903558	3.85697E-08
mmu04066	HIF-1 signaling pathway	2.85471E-08	24.51723547	5.41206E-08
mmu05140	Leishmaniasis	3.12912E-08	36.46938776	5.81123E-08
mmu05168	Herpes simplex virus 1 infection	3.33197E-08	9.351125065	6.06419E-08
mmu04151	PI3K-Akt signaling pathway	3.66484E-08	10.99077439	6.53922E-08

Term	pathway	PValue	Fold Enrichment	FDR
mmu04920	Adipocytokine signaling pathway	4.03808E-08	34.97064579	7.06664E-08
mmu04380	Osteoclast differentiation	5.55148E-08	22.27138184	9.40156E-08
mmu05212	Pancreatic cancer	5.57894E-08	33.15398887	9.40156E-08
mmu05133	Pertussis	6.51427E-08	32.31464738	1.07781E-07
mmu05135	Yersinia infection	7.18709E-08	21.45258103	1.1679E-07
mmu05235	PD-L1 expression and PD-1 checkpoint pathway in cancer	1.2472E-07	29.00974026	1.99114E-07
mmu04024	cAMP signaling pathway	1.31854E-07	14.65287901	2.06875E-07
mmu05134	Legionellosis	6.51126E-07	35.29295589	1.00428E-06
mmu04062	Chemokine signaling pathway	8.34126E-07	14.9618001	1.26509E-06
mmu04919	Thyroid hormone signaling pathway	9.55655E-07	20.5875576	1.42565E-06
mmu04010	MAPK signaling pathway	1.06709E-06	11.12625389	1.56621E-06
mmu04622	RIG-I-like receptor signaling pathway	1.28665E-06	30.81920092	1.85849E-06
mmu04115	p53 signaling pathway	1.47839E-06	29.97483925	2.10208E-06
mmu05223	Non-small cell lung cancer	1.58234E-06	29.56977386	2.21527E-06
mmu05220	Chronic myeloid leukemia	2.05713E-06	28.0533752	2.83635E-06
mmu01521	EGFR tyrosine kinase inhibitor resistance	2.19173E-06	27.69826918	2.97682E-06
mmu04662	B cell receptor signaling pathway	2.63752E-06	26.68491787	3.52962E-06
mmu05224	Breast cancer	2.90325E-06	17.01904762	3.82892E-06
mmu04012	ErbB signaling pathway	3.53609E-06	25.1513019	4.59692E-06
mmu05171	Coronavirus disease - COVID-19	4.24056E-06	11.71707237	5.43508E-06
mmu04658	Th1 and Th2 cell differentiation	4.65927E-06	23.78438332	5.8888E-06
mmu05020	Prion disease	7.21212E-06	10.80574452	8.99045E-06
mmu04064	NF-kappa B signaling pathway	8.91314E-06	20.83965015	1.09607E-05
mmu04935	Growth hormone synthesis, secretion and action	1.57346E-05	18.54375649	1.90913E-05
mmu05206	MicroRNAs in cancer	1.66025E-05	9.503423519	1.98793E-05
mmu05213	Endometrial cancer	1.7474E-05	31.43912738	2.03864E-05
mmu04370	VEGF signaling pathway	1.7474E-05	31.43912738	2.03864E-05
mmu05415	Diabetic cardiomyopathy	2.2771E-05	11.87375415	2.62298E-05
mmu04726	Serotonergic synapse	2.90763E-05	16.32957661	3.30743E-05
mmu04728	Dopaminergic synapse	3.588E-05	15.62973761	4.03096E-05
mmu04910	Insulin signaling pathway	3.84025E-05	15.40960046	4.26174E-05
mmu04140	Autophagy - animal	4.38586E-05	14.98741963	4.8086E-05
mmu05226	Gastric cancer	5.15025E-05	14.49114745	5.57944E-05
mmu05012	Parkinson disease	7.46821E-05	9.597207304	7.99538E-05

Term	pathway	PValue	Fold Enrichment	FDR
mmu04630	JAK-STAT signaling pathway	9.2885E-05	12.79627641	9.82853E-05
mmu04211	Longevity regulating pathway	9.88183E-05	20.26077098	0.000103362
mmu05231	Choline metabolism in cancer	0.000148696	18.23469388	0.000153766
mmu04510	Focal adhesion	0.00021212	10.72629052	0.000216887
mmu05146	Amoebiasis	0.000214665	16.57699443	0.00021705
mmu05030	Cocaine addiction	0.000288973	30.39115646	0.000288973
**mmu00330**	**Arginine and proline metabolism**	**0.000409757**	**27.0143613**	**0.000409757**
mmu05014	Amyotrophic lateral sclerosis	0.000458446	6.862519201	0.000458446
mmu04923	Regulation of lipolysis in adipocytes	0.000505911	25.1513019	0.000505911
mmu04371	Apelin signaling pathway	0.000509022	13.21354629	0.000509022
mmu05321	Inflammatory bowel disease	0.000706801	22.44270016	0.000706801
mmu04921	Oxytocin signaling pathway	0.000787358	11.76431863	0.000787358
mmu04137	Mitophagy - animal	0.000841451	21.14167406	0.000841451
mmu05031	Amphetamine addiction	0.000841451	21.14167406	0.000841451
mmu05211	Renal cell carcinoma	0.000914468	20.54613395	0.000914468
mmu05221	Acute myeloid leukemia	0.000914468	20.54613395	0.000914468
mmu05225	Hepatocellular carcinoma	0.001235737	10.41982507	0.001235737
mmu04022	cGMP-PKG signaling pathway	0.001315698	10.24421004	0.001315698
mmu05016	Huntington disease	0.00131614	7.174305788	0.00131614
mmu04914	Progesterone-mediated oocyte maturation	0.001931544	15.85625555	0.001931544
mmu04912	GnRH signaling pathway	0.001931544	15.85625555	0.001931544
mmu05202	Transcriptional misregulation in cancer	0.003085805	8.10430839	0.003085805
mmu04014	Ras signaling pathway	0.003443528	7.859781844	0.003443528
mmu04725	Cholinergic synapse	0.003549135	12.79627641	0.003549135
mmu04650	Natural killer cell mediated cytotoxicity	0.003817888	12.46816675	0.003817888
mmu04611	Platelet activation	0.004908239	11.39668367	0.004908239
mmu05143	African trypanosomiasis	0.005419135	26.68491787	0.005419135
mmu04068	FoxO signaling pathway	0.005690459	10.80574452	0.005690459
mmu04550	Signaling pathways regulating pluripotency of stem cells	0.006543527	10.27306697	0.006543527
mmu04930	Type II diabetes mellitus	0.007666548	22.32819658	0.007666548
**mmu00380**	**Tryptophan metabolism**	**0.00924984**	**20.26077098**	**0.00924984**
mmu01100	Metabolic pathways	0.00993117	2.470217151	0.00993117
mmu04934	Cushing syndrome	0.010026841	8.787804278	0.010026841
mmu04530	Tight junction	0.010524774	8.631807753	0.010524774
mmu04310	Wnt signaling pathway	0.011210755	8.432228383	0.011210755
mmu04340	Hedgehog signaling pathway	0.011324909	18.23469388	0.011324909
mmu04141	Protein processing in	0.011741887	8.288497217	0.011741887

Term	pathway	PValue	Fold Enrichment	FDR
	endoplasmic reticulum			
mmu04913	Ovarian steroidogenesis	0.012432228	17.36637512	0.012432228
mmu04623	Cytosolic DNA-sensing pathway	0.012811479	17.09502551	0.012811479
mmu04664	Fc epsilon RI signaling pathway	0.013979265	16.32957661	0.013979265
mmu00232	Caffeine metabolism	0.016010784	121.5646259	0.016010784
mmu05218	Melanoma	0.016875664	14.78488693	0.016875664
mmu05323	Rheumatoid arthritis	0.022864412	12.57565095	0.022864412
**mmu00590**	**Arachidonic acid metabolism**	**0.024354042**	**12.15646259**	**0.024354042**
mmu05416	Viral myocarditis	0.024859255	12.02287508	0.024859255
mmu04020	Calcium signaling pathway	0.0277106	5.978588157	0.0277106
mmu04928	Parathyroid hormone synthesis, secretion and action	0.034083886	10.13038549	0.034083886
mmu04670	Leukocyte transendothelial migration	0.040040276	9.271878243	0.040040276
mmu04060	Cytokine-cytokine receptor interaction	0.044670271	4.94500173	0.044670271
mmu04750	Inflammatory mediator regulation of TRP channels	0.046360102	8.547512755	0.046360102
mmu04152	AMPK signaling pathway	0.048984697	8.288497217	0.048984697

2.3. Result of Metabolites

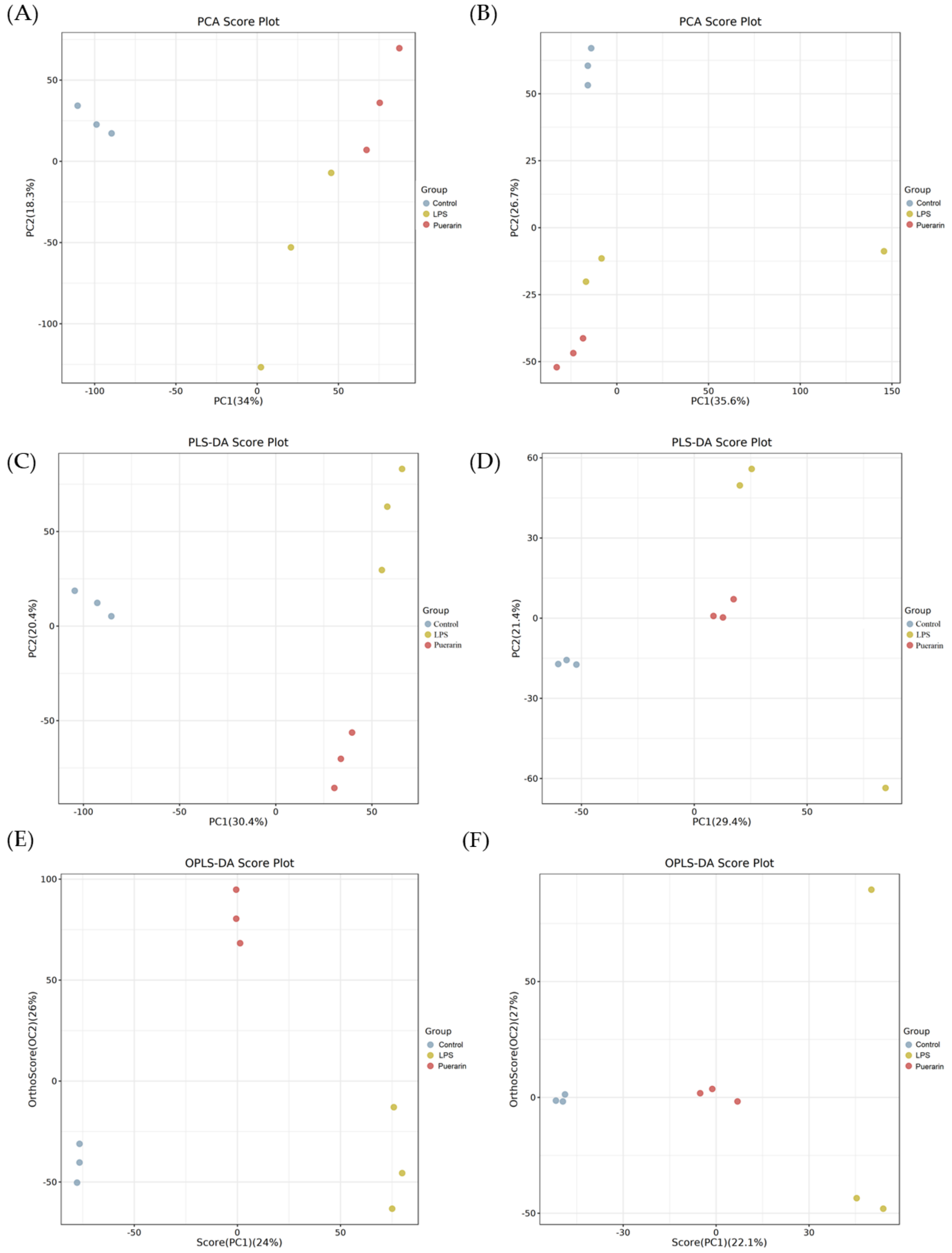

Supplement Figure S1: The PCA score plots of samples in positive ion mode (A) and in negative ion mode (B); The PLS-DA score plots of samples in positive ion mode (C) and in negative ion mode (D); The OPLS-DA score plots of samples in positive ion mode (E) and in negative ion mode (F).

Supplement Table S3: The KEGG pathways enriched by Metabolites analysis

Category	KEGG pathway	Pvalue	Expected
Amino acid metabolism	Phenylalanine, tyrosine and tryptophan biosynthesis	0.14315	0.15139
	Valine, leucine and isoleucine biosynthesis	0.26611	0.30279
	Taurine and hypotaurine metabolism	0.26611	0.30279
	Phenylalanine metabolism	0.3717	0.45418
	**Arginine and proline metabolism**	**0.42596**	**1.4382**
	Aminoacyl-tRNA biosynthesis	0.55058	1.8167
	Lysine degradation	0.62187	0.94622
	Alanine, aspartate and glutamate metabolism	0.6639	1.0598
	Valine, leucine and isoleucine degradation	0.79072	1.5139
	**Tryptophan metabolism**	**0.79886**	**1.5518**
	Selenocompound metabolism	0.54007	0.75697
	Glutathione metabolism	0.6639	1.0598
Carbohydrate metabolism	Glyoxylate and dicarboxylate metabolism	0.34353	1.2112
	Amino sugar and nucleotide sugar metabolism	0.41255	1.4004
	Fructose and mannose metabolism	0.50268	0.68127
	Citrate cycle (TCA cycle)	0.54007	0.75697
	Pentose phosphate pathway	0.57469	0.83267
	Pyruvate metabolism	0.57469	0.83267
	Propanoate metabolism	0.59102	0.87052
	Glycolysis / Gluconeogenesis	0.63642	0.98406
Lipid metabolism	Ether lipid metabolism	0.54007	0.75697
	Sphingolipid metabolism	0.55771	0.79482
	**Arachidonic acid metabolism**	**0.75481**	**1.3625**
	Glycerophospholipid metabolism	0.75481	1.3625
	Primary bile acid biosynthesis	0.8351	1.741
Metabolism of cofactors and vitamins	Nicotinate and nicotinamide metabolism	0.016926	0.56773
	Folate biosynthesis	0.65042	1.0219
	Porphyrin and chlorophyll metabolism	0.68933	1.1355
Nucleotide metabolism	Pyrimidine metabolism	0.18072	1.4761
	Purine metabolism	0.46083	2.498
